# Serial Intracranial Flow Rate Measurements Using Quantitative Magnetic Resonance Angiography Following Large-Vessel Occlusion Stroke

**DOI:** 10.3390/brainsci16020171

**Published:** 2026-01-31

**Authors:** Jean-Philippe Dufour, Corinne Inauen, Lara Höbner, Jacopo Bellomo, Tilman Schubert, Martina Sebök, Jorn Fierstra, Elisa Colombo, Christiaan Hendrik Bas van Niftrik, Marco Piccirelli, Christoph Globas, Zsolt Kulcsar, Andreas Luft, Susanne Wegener, Luca Regli, Giuseppe Esposito

**Affiliations:** 1 Department of Neurosurgery, Clinical Neuroscience Center, University Hospital Zurich, University of Zurich, 8091 Zurich, Switzerland; jean-philippe.dufour@usz.ch (J.-P.D.); lara.hoebner@usz.ch (L.H.); jacopo.bellomo@usz.ch (J.B.); martina.seboek@usz.ch (M.S.); jorn.fierstra@usz.ch (J.F.); elisa.colombo@usz.ch (E.C.); bas.vanniftrik@usz.ch (C.H.B.v.N.); luca.regli@usz.ch (L.R.); 2 Department of Neurology, Clinical Neuroscience Center, University Hospital Zurich, University of Zurich, 8091 Zurich, Switzerland; corinne.inauen@usz.ch (C.I.); christoph.globas@usz.ch (C.G.); andreas.luft@usz.ch (A.L.); susanne.wegener@usz.ch (S.W.); 3 Department of Neuroradiology, Clinical Neuroscience Center, University Hospital Zurich, University of Zurich, 8091 Zurich, Switzerland; tilman.schubert@usz.ch (T.S.); marco.piccirelli@usz.ch (M.P.); zsolt.kulcsar@usz.ch (Z.K.)

**Keywords:** stroke, ischaemic stroke, magnetic resonance angiography, thrombectomy, cerebrovascular circulation, cerebrovascular disorders, haemodynamics

## Abstract

**Background/Objectives**: Haemodynamic changes following ischaemic large-vessel occlusion (LVO) stroke might affect clinical outcome, including after endovascular recanalization. Using non-invasive quantitative MRA (qMRA), we report for the first time serial intracranial flow rate measurements following LVO and investigate flow rate changes, collateral pathway development, and their possible clinical significance. **Methods**: We report data from the prospective IMPreST study (Interplay of Microcirculation and Plasticity after Ischemic Stroke, registered at clinicaltrials.gov, no. NCT04035746). Patients with first-ever unilateral internal carotid artery (ICA) and/or M1/2 middle cerebral artery (MCA) occlusions were included. After being evaluated for gold-standard treatment, including endovascular thrombectomy, patients underwent early (<3 days) and late subacute (7 ± 3 days) qMRA flow measurements of the M1-MCA, A2-ACA (anterior cerebral artery), and P2-PCA (posterior cerebral artery) segments bilaterally. **Results**: Among 31 patients enrolled, 23 patients (17 recanalized, 6 non-recanalized) received both qMRA sessions. M1 volume flow rate (VFR) ratios (ischaemic/non-ischaemic hemisphere) in recanalized patients were symmetric (0.98–1.01) over time, while in non-recanalized patients M1 VFR ratios remained lower (0.74–0.77). P2 VFR ratios increased over time and were negatively correlated with M1 VFR ratios in late measurements (*p* = 0.016), possibly reflecting subacute activation of leptomeningeal collaterals via P2-PCA. In recanalized patients, lower M1 VFR ratios at 7 ± 3 days were significantly associated with a higher NIHSS at discharge after adjusting for infarct size, age, and NIHSS at admission (*p* = 0.02). Total hemispheric flow significantly decreased by up to 9% between early and late measurements in both the ischaemic and non-ischaemic hemispheres (*p* = 0.005). **Conclusions**: qMRA may help to understand flow status and collateral pathway activation after LVO stroke. Past the acute phase, low arterial flow to the affected region is associated with poorer neurological status, including after successful recanalization.

## 1. Introduction

Intracranial large-vessel occlusion (LVO), i.e., occlusion of the internal carotid artery (ICA) or middle cerebral artery (MCA), leads to acute ischaemia of the affected brain regions with potentially devastating consequences [1]. The standard of care comprises endovascular thrombectomy (EVT) with preceding intravenous thrombolysis, aiming to restore distal blood flow [2]. However, clinical outcomes after successful recanalization vary greatly, and many patients experience unfavourable neurological outcomes despite timely intervention [3,4]. Recent work has shown that even after recanalization, perfusion of the ischaemic region is mediated by a dynamic, complex process involving autoregulation, inflammation, microcirculatory changes and the development of collateral pathways [5,6,7]. This process may result in local, pathological hypo- or hyperperfusion, and the ensuing haemodynamic status might critically affect clinical outcomes [8]. Nevertheless, how arterial flow rates develop after LVO stroke remains largely unknown.

Quantitative MR Angiography (qMRA), combining 2D phase-contrast imaging with time-of-flight (TOF) MRA 3D reconstructions for optimal placement of the measuring planes, is an increasingly utilized technique allowing for accurate and non-invasive measurements of absolute volumetric flow rates (VFRs, in mL/min). Age- and sex-adjusted VFR ranges of the larger intracranial arteries are available [9], and qMRA values have been shown to correlate highly with those measured invasively using sonographic flow meters [10]. To date, qMRA has been used in a wide variety of cerebrovascular pathologies, including to assess cerebral hyperperfusion syndrome after carotid endarterectomy [11], to guide patient selection for urgent extracranial–intracranial bypass in acute stroke [5,12], to predict stroke risk in patients with extracranial arteriosclerotic stenosis [13,14,15,16], and to analyse VFR changes following revascularization in chronic steno-occlusive disease [17,18,19,20]. In this prospective study, we use serial qMRA imaging to assess VFR changes in the anterior, middle and posterior cerebral arteries bilaterally following acute LVO stroke in both recanalized and non-recanalized patients. Our aim is to provide an exploratory analysis of intracranial VFR developments after LVO and to explore their possible relationship with neurological outcome.

## 2. Materials and Methods

### 2.1. Patient Inclusion and Study Protocol

This study reports data from patients admitted to the University Hospital of Zurich with acute ischaemic stroke following unilateral large-vessel occlusion of the anterior circulation and who received qMRA as part of the IMPreST study (Interplay of Microcirculation and Plasticity after Ischemic Stroke, registered at clinicaltrials.gov, no. NCT04035746). This study was a prospective cohort study running from October 2019 to March 2022, aiming at analysing the role of advanced imaging modalities in assessing changes in microcirculation and associated clinical outcomes following acute LVO stroke. As reported previously [21], patient inclusion criteria were (a) ≤72 h first-ever clinical ischaemic stroke at hospital admission; (b) unilateral occlusion of the M1/M2-segment of the MCA and/or intracranial ICA, and perfusion deficits with cortical involvement; (c) ≥18 years; (d) independent living before stroke (modified Ranking Scale (mRS) ≤ 3); (e) written informed consent of the patient, or the written authorization of an independent doctor who is not involved in the research and obtainment of post hoc written informed consent from the patient/next of kin. Exclusion criteria were (a) major cardiac, psychiatric, or neurological diseases; (b) early seizures; (c) known or suspected non-compliance, drug or alcohol abuse; (d) contraindications for MRI; (e) documented evidence that the patient does not wish to participate in a scientific study.

After being considered for and possibly undergoing EVT, patients received a series of standardized MRI protocols at predefined time points (≤72 h and 7 ± 3 days) from stroke onset. Imaging included diffusion-weighted imaging (DWI), 3D TOF-MRA and 2D phase-contrast qMRA using non-invasive optimal vessel analysis (NOVA) software (VasSol Inc., River Forest, IL, USA). Demographic, clinical and anatomical variables were collected, including cardiovascular risk factors, clinical outcome using the modified Rankin scale (mRS) and National Institute of Health Stroke Scale (NIHSS), angio-anatomy of the circle of Willlis, and DWI lesion volume. Stroke aetiology was reported as Trial of Org 10172 in Acute Stroke Treatment (TOAST) classification. Collateralization on DSA for patients undergoing ETV was assessed using the American Society of Interventional and Therapeutic Neuroradiology/Society of Interventional Radiology (ASITN/SIR) scale, where possible.

### 2.2. Image Acquisition and Processing

Blood flow quantification using qMRA has been described in detail previously and was performed using the commercially available NOVA software [9]. We measured the volumetric flow rate (VFR, in mL/min) of the following six vessels: the first segment of the middle cerebral artery (M1-MCA), the second segment of the anterior cerebral artery (A2-ACA), and the second segment of the posterior cerebral artery (P2-PCA), all bilaterally. This enabled us to calculate arterial territory-specific flow as well as total hemispheric flow.

### 2.3. Statistics

Statistical analysis was done using Python version 3.9.13 and Jamovi version 2.3.28. Statistical significance was assumed at *p* < 0.05 throughout. Continuous variables are reported as means ± standard deviations (SDs). Ordinal variables are reported as medians ± inter-quartile ranges (IQRs) and dichotomous variables as frequencies. The distributions of baseline parameters between the recanalized and non-recanalized patients were compared using the Chi-squared test, two-tailed Welch’s *t*-test, or the Mann–Whitney U test, as appropriate. VFR ratios of the ischaemic/non-ischaemic hemispheres were calculated within each patient separately. Absolute VFR and VFR ratios over time were analysed using linear mixed effects models: scanning session (early vs. late), recanalization status, hemisphere (for absolute flow) and their interactions were treated as fixed effects, while patients were treated as random effects with random intercepts and within-patient hemispheric flow differences (slopes). Assumptions (linearity, normality of residuals, and homoscedasticity) were visually inspected for each model iteration. The significance of fixed effects was assessed using the t-statistic with Satterthwaite’s degrees-of-freedom approximation, as available through the lmerTest package version 3.2. Model equations and detailed parameter estimations are given in the Supplementary Materials. The relationship between VFR and neurological outcome, as measured on the National Institute of Health Stroke Scale (NIHSS), was assessed using multiple linear regression, including age, NIHSS at admission and infarct core size (calculated using DWI lesion volumes) as covariates. Note that using an ordinal logistic regression model did not change the qualitative nature of the results; however, the proportional odds assumption was not satisfied.

### 2.4. Ethics

The IMPreST prospective cohort study was approved by the research ethics committee of the Canton of Zurich (Kantonale Ethikkommission Zürich, KEK-Nr. 2019-00750), in accordance with the Declaration of Helsinki. In addition, informed consent was obtained from each patient before inclusion in the study, and all patient information was de-identified.

## 3. Results

### 3.1. Patient Characteristics and Recanalization

**Figure 1:**
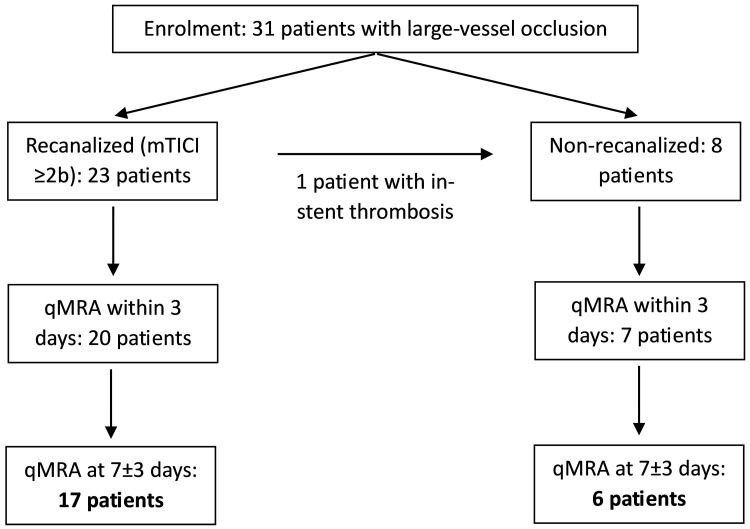
Patient inclusion and study flowchart.

Thirty-one patients enrolled in the IMPreST study underwent NOVA qMRA as part of their follow-up, with 23 patients included in both the first (<3 days from stroke, mean: 40 h) and the second study (7 ± 3 days from stroke, mean: 5.9 days; Figure 1). Four patients each did not receive the first or the second qMRA study. The mean age was 65.6 ± 14.3 years, with 11 patients being male.

Recanalization via EVT was successfully performed in 23 patients (mTICI grade ≥ 2b, symptom onset-to-groin puncture: 267 ± 191 min), while 8 patients were not recanalized. Of these non-recanalized patients, 7 did not undergo either intravenous lysis or EVT, due to spontaneous improvement of symptoms and/or an elapsed time window. One patient initially received EVT, including ICA stenting; however, ultrasound imaging performed the same day showed in-stent thrombosis—thus, the patient was classified as non-recanalized.

All recanalized patients presented with M1/M2 occlusions or tandem ICA-MCA occlusions, whereas all non-recanalized patients presented with ICA occlusions but patent MCAs, except for one patient with tandem ICA-MCA occlusion. The clinical and demographic factors for both recanalized and non-recanalized patients are shown in Table 1. Note that patients who did not undergo recanalization had significantly smaller infarction volumes and better clinical admission scores (NIHSS and mRS), suggesting an already favourable collateral flow profile at the time of stroke, likely due to acute-on-chronic vessel occlusions. This was also reflected in the stroke aetiologies: there were no cardioembolic (TOAST 2) strokes in the non-recanalized group, whereas this was the most frequent aetiology in recanalized patients.

### 3.2. M1 Segments

**Figure 2:**
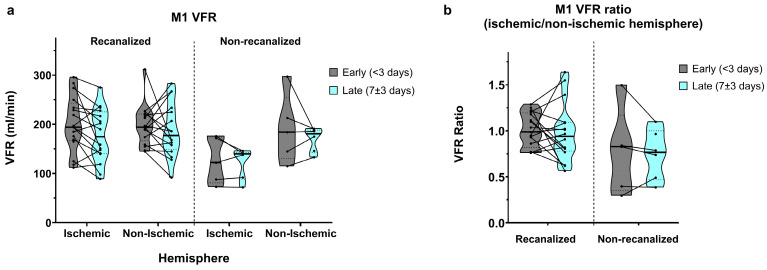
(**a**) M1 segment volumetric flow rates (VFRs) in early (<3 days) and late (7 ± 3 days) measurements, in recanalized and non-recanalized patients. (**b**) M1 VFR ratios (ischaemic/non-ischaemic hemisphere) in early and late measurements, in recanalized and non-recanalized patients.

In recanalized patients, early (<3 days) M1 VFR measurements in the ischaemic and non-ischaemic hemispheres were comparable (201.2 ± 55.8 vs. 198.8 ± 40.1 mL/min, average ischaemic/non-ischaemic hemisphere ratio of 1.01 ± 0.18; Figure 2a,b). In late (7 ± 3 days) measurements, M1 VFR dropped bilaterally (177.1 ± 54.2 vs. 188.1 ± 55.0 mL/min, average ischaemic/non-ischaemic hemisphere ratio of 0.98 ± 0.31).

In non-recanalized patients, M1 VFR in the ischaemic hemisphere remained low compared to both recanalized patients and to the non-ischaemic hemisphere, in early measurements (126.2 ± 47.1 vs. 190.8 ± 70.1 mL/min, average ratio of 0.77 ± 0.47) as well as in late measurements (121.7 ± 31.5 vs. 169.7 ± 24.1 mL/min, average ratio of 0.74 ± 0.27). Of note, due to concurrent M1 stenosis of the non-ischaemic hemisphere, one non-recanalized patient with ICA occlusion showed an ischaemic/non-ischaemic M1 VFR ratio > 1.

The mixed effects model showed a significant interaction between recanalization status and hemisphere on M1 VFR (*p* = 0.034). Although average M1 VFR dropped universally between early and late measurements, the main effect of time did not reach significance (*p* = 0.08), with no interaction between time and recanalization status. Finally, while ischaemic/non-ischaemic M1 VFR ratios were lower in non-recanalized patients, the main effect of recanalization on M1 VFR ratios was not significant (*p* = 0.075), likely due to the non-recanalized outlier with ratio >1 mentioned above (*p* < 0.01 after its exclusion).

### 3.3. P2 Segments

**Figure 3:**
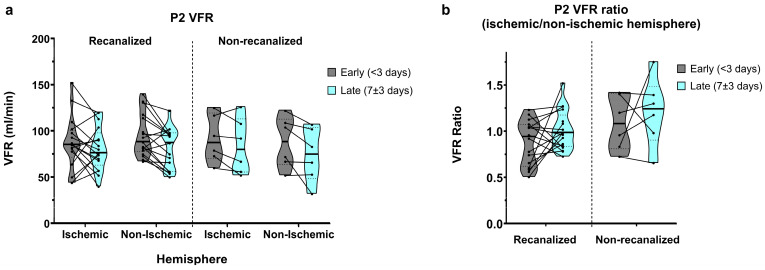
(**a**) P2-segment VFR in early and late measurements, for recanalized and non-recanalized patients. (**b**) P2 VFR (ischaemic/non-ischaemic hemisphere) ratios in early and late measurements, for recanalized and non-recanalized patients.

In recanalized patients, average P2 VFRs in early measurements showed a relative dominance of the non-ischaemic hemisphere (86.7 ± 28.5 vs. 95.4 ± 23.4 mL/min, average ratio of 0.94 ± 0.33; Figure 3a,b). P2 VFRs decreased bilaterally in late measurements, yet this decrease was more pronounced in the non-ischaemic hemisphere, resulting in a symmetric ischaemic/non-ischaemic P2 VFR ratio (80.6 ± 23.5 vs. 80.9 ± 22.0 mL/min, average ratio of 1.01 ± 0.21).

Non-recanalized patients, by contrast, showed relative P2 VFR dominance in the ischaemic hemisphere in early measurements (91.5 ± 25.6 vs. 87.7 ± 27.7 mL/min, average ratio of 1.09 ± 0.29). In late measurements, P2 VFR also decreased bilaterally on average, though this reduction was less pronounced in the ischaemic hemisphere (83.5 ± 29.9 vs. 73.8 ± 29.0 mL/min), resulting in a further increased P2 VFR ratio of 1.21 ± 0.37 in the later measurements.

Overall, P2 VFR showed a significant decrease between the early and late measurements (main effect of time: *p* = 0.003), with no interaction between recanalization and time. P2 VFR ratios did not show significant effects of time, recanalization status, or their interaction.

**Figure 4:**
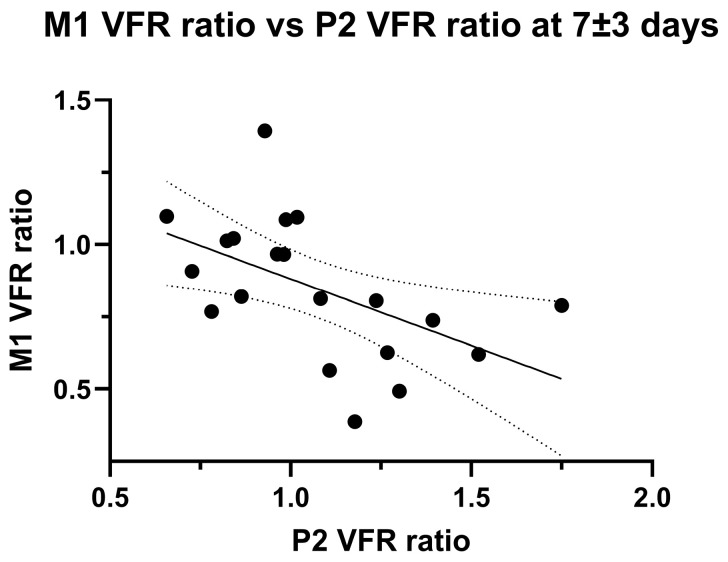
Linear negative correlation between M1 VFR ratio and P2 VFR ratio in late (7 ± 3 days) measurements. Note that two outliers were excluded (>third quartile + 1.5 × inter-quartile range, *p* = 0.07 with outlier inclusion).

The development of P2 VFR hemispheric asymmetry might represent leptomeningeal collateral activation from insufficient primary collateral flow. Accordingly, we found a significant negative linear correlation between ischaemic/non-ischaemic hemispheric M1 VFR ratios and P2 VFR ratios at the second imaging timepoint (*p* = 0.016, Pearson’ R = 0.53; Figure 4).

### 3.4. A2 Segments

**Figure 5:**
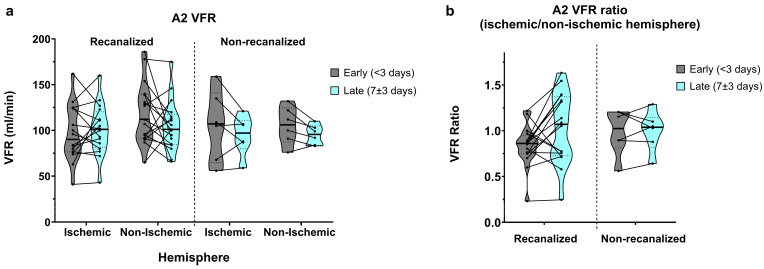
(**a**) A2-segment VFR in early (<3 days) and late (7 ± 3 days) measurements, in recanalized and non-recanalized patients. (**b**) A2 VFR ratios (ischaemic/non-ischaemic hemisphere) in early and late measurements, in recanalized and non-recanalized patients.

Flow in the A2 segments was relatively widely spread, as expected from the anatomical variation found in the anterior communicating artery (AComm) complex. In recanalized patients, A2 VFR in early measurements was on average higher in the non-ischaemic hemisphere (105.1 ± 45.7 vs. 118.8 ± 33.5 mL/min, average ratio of 0.94 ± 0.49; Figure 5a,b) and dropped in the non-ischaemic hemisphere on the second measurement (109 ± 42.7 vs. 105.2 ± 27.9 mL/min, average ratio of 1.13 ± 0.57). In contrast, non-recanalized patients showed decreasing yet symmetric A2 flow between the early (105.3 ± 27.7 vs. 105.5 ± 20.6 mL/min, average ratio of 0.99 ± 0.25) and late (94.7 ± 21.6 vs. 95.2 ± 10.7 mL/min, average ratio of 1.0 ± 0.22) measurements. No statistically significant effects of hemisphere, recanalization status, or time were seen on either the absolute A2 VFR or A2 VFR ratios.

### 3.5. Hemispheric Flow

**Figure 6:**
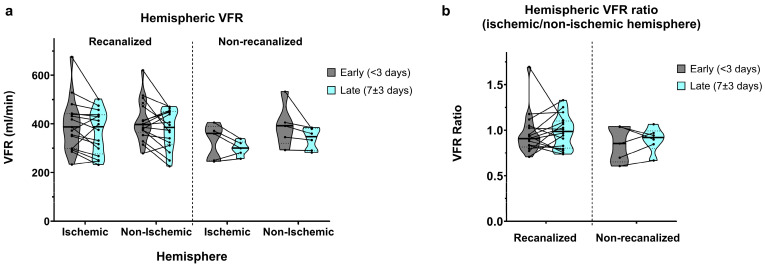
(**a**) Hemispheric VFR in early (<3 days) and late (7 ± 3 days) measurements, in recanalized and non-recanalized patients. (**b**) Hemispheric VFR ratios (ischaemic/non-ischaemic hemisphere) in early and late measurements, in recanalized and non-recanalized patients.

Total hemispheric VFR (A2 + M1 + P2) displayed similar aspects to M1 segmental VFR. In recanalized patients, early measurements showed slightly lower flow in the ischaemic hemisphere (388.6 ± 106.5 vs. 412.9 ± 84.8 mL/min, average ratio of 0.95 ± 0.22; Figure 6a,b). Late measurements showed a bilateral flow decrease with nearly symmetric VFR (367.0 ± 89.0 vs. 375.8 ± 79.6 mL/min, average ratio of 0.99 ± 0.18).

As expected, non-recanalized patients showed more pronounced ischaemic vs. non-ischaemic hemispheric asymmetry in the early measurements (326.8 ± 73.9 vs. 394.0 ± 89.4 mL/min, average ratio of 0.85 ± 0.19). Late measurements showed a similar bilateral VFR decrease, together with an improvement in hemispheric VFR symmetry (299.6 ± 28.7 vs. 338.7 ± 44.3 mL/min, average ratio of 0.9 ± 0.13).

Correspondingly, we found a significant main effect of time (*p* = 0.005) on hemispheric VFR, with no significant interaction between time and recanalization or between time and hemisphere. The main effect of hemisphere on hemispheric VFR remained barely above statistical significance (*p* = 0.051), with no significant interaction between recanalization and hemisphere. Finally, we did not find significant effects of time, recanalization or their interaction on hemispheric VFR ratios.

### 3.6. qMRA and Clinical Outcome

We further investigated whether the intracranial flow status was related to radiological or clinical outcome. Firstly, we analysed any correlation between infarct volume in DWI sequences and qMRA VFR in recanalized patients. We found no significant correlation between infarct size and any segmental or hemispheric VFR ratio in the first or second qMRA measurements, or between infarct size and changes in flow or flow ratios over time. Likewise, there was no significant association between any flow metrics and haemorrhagic transformation.

**Figure 7:**
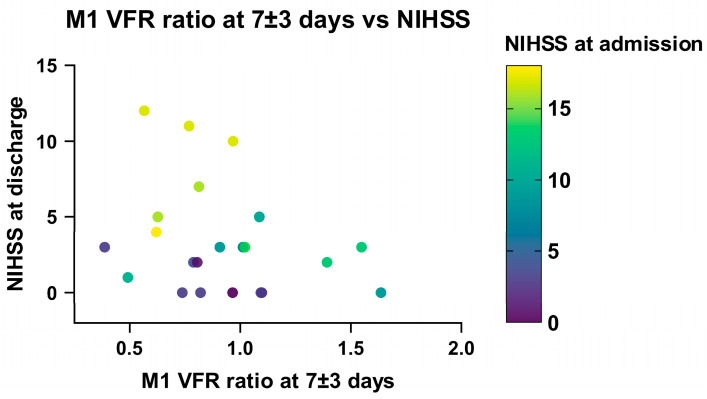
Relationship between NIHSS at discharge, NIHSS at admission and M1 VFR ratio in late measurements (7 ± 3 days) in recanalized patients.

Linear regression analysis in recanalized patients showed a significant association between a low ischaemic/non-ischaemic M1 VFR ratio in the late (7 ± 3 days) measurement and a higher NIHSS score at discharge (*p* = 0.047). This relationship remained significant when correcting for NIHSS on admission, DWI infarction volume and age (*p* = 0.02; Supplementary Table S1, Figure 7), as well as when including non-recanalized patients (*p* = 0.035). Of note, using the change in M1 VFR ratios or total hemispheric VFR ratios between the early and late qMRA measurements led to essentially the same results.

The correlation between these flow metrics and NIHSS at 90 days follow-up no longer reached statistical significance (*p* = 0.079 for change in M1 VFR ratio), while NIHSS at admission remained significantly correlated with longer-term follow-up (*p* = 0.03). Moreover, M1 or hemispheric VFR ratios in the early measurements (<3 days) alone were not significantly associated with clinical outcomes at any timepoint. Finally, high ischaemic/non-ischaemic P2 VFR ratios or high P2/M1 VFR ratios on the ischaemic hemisphere—as a potential sign of leptomeningeal collateral activation—were not statistically correlated with neurological outcome.

## 4. Discussion

In this study, patients with LVO stroke underwent serial qMRA-NOVA imaging after receiving optimal treatment, with our analysis concentrating on patients receiving imaging in both the early (<3 days) and late subacute (7 ± 3 days) setting. By including the three arterial segments M1, A2 and P2 bilaterally, we assessed the temporal development of regional and global flow. To the best of our knowledge, no other study has reported serial intracranial flow rate measurements following LVO stroke. Our exploratory analysis revealed a number of findings: (i) qMRA accurately reflected failed reperfusion in patients not undergoing recanalization in the form of persistently low M1 VFR to the affected region; (ii) global intracranial flow significantly decreased globally between the early and late measurements across all patients; (iii) P2 flow showed a relative increase in the ischaemic versus the non-ischaemic hemispheres between early and late measurements, thereby also correlating with lower relative M1 flow in late measurements; and (iv) low relative M1 flow at 7 ± 3 days post-stroke was associated with poor neurological outcome at discharge, including in patients successfully recanalized.

### 4.1. Flow Decrease over Time

The decrease in arterial flow rates over time was evident across all arterial territories, including the posterior circulation (P2 segment) of the unaffected hemisphere, as well as in both recanalized and non-recanalized patients. Our results possibly tie in with previous reports of transhemispheric diaschisis after stroke [22,23,24], i.e., the reduction in metabolic activity and cerebral blood flow (CBF) in a structurally intact brain region that is distant from, but anatomically connected to, the site of an acute stroke. The pathophysiological mechanisms and relevance of this phenomenon remain unclear. It is also possible that this flow reduction reflects the normalization of an acute, brain-wide relative hyperperfusion in response to local ischaemia, mirroring the previously reported acute hyperperfusion following traumatic brain injury [25,26,27,28]. A transient impairment of cerebral autoregulation, as described following ischaemic stroke, may plausibly contribute [29,30]. However, we cannot exclude systemic factors—such as elevated blood pressure in the acute phase—influencing global cerebral blood flow, although we found no association between last measured pre-qMRA blood pressure and VFR measurements.

### 4.2. Stroke Aetiologies in Recanalized vs. Non-Recanalized Patients

It is important to note that stroke presentations and aetiologies differed between recanalized and non-recanalized patients. Indeed, patients who did not undergo recanalization (due to elapsed time windows, or mild or resolving symptoms) all presented with ICA occlusions, significantly smaller infarct sizes, fewer symptoms, and large-artery atherosclerosis (TOAST 1) or undetermined (TOAST 5) stroke aetiologies. By contrast, recanalized patients presented with MCA or tandem ICA-MCA occlusions, with cardioembolic (TOAST 2) aetiologies being the most frequent. Hence, the symptomatic ICA occlusions in non-recanalized patients most likely occurred secondary to chronic, high-grade atherosclerotic ICA stenosis, and thus with collateral pathways already at least partially activated prior to stroke. Indeed, P2 flow ratios were higher and already >1 on average in early imaging of non-recanalized patients, possibly reflecting an activation of posterior leptomeningeal collaterals. These pathophysiological differences clearly limit direct comparisons between both groups and impact the interpretation of our results, as stated below.

### 4.3. Posterior Circulation Flow Increase

A growing line of evidence suggests that the activation of secondary collaterals, particularly leptomeningeal collaterals, is indicative of a reduced or even exhausted cerebrovascular reserve [31,32,33]. Furthermore, qMRA has previously been used to assess posterior leptomeningeal collaterals by showing significantly increased ipsilateral P2 flow in patients with chronically reduced unilateral ICA or MCA flow, with ipsilateral/contralateral P2 flow ratios in several cases >2 [34]. Accordingly, we show a relative increase over time in P2 segment flow in the ischaemic hemisphere compared to the non-ischaemic hemisphere, especially in non-recanalized patients. The significant negative correlation between M1 and P2 VFR ratios in late measurements further point towards a compensatory mechanism for persistent inadequate affected tissue perfusion (i.e., low M1 flow). Nevertheless, the change in P2 flow ratio over time was not statistically significantly higher in non-recanalized vs. recanalized patients, contrary to expectations. This might be partly explained by the lower number of non-recanalized patients and unbalanced group sizes, or due to the time window between imaging sessions being too short to capture sufficient activation of posterior collaterals. An additional confounding factor is the abovementioned, likely acute-on-chronic occlusion nature in the non-recanalized group, with the relative increase in ipsilateral P2 flow post-stroke therefore possibly attenuated by pre-existing vertebrobasilar recruitment. Thus, our results remain indirect and require further confirmation in longitudinal studies.

Finally, while a high P2 VFR ratio or low M1/P2 ratio (as markers of exhausted cerebrovascular reserve) did not correlate significantly with neurological outcome in our study, additional work should be done to elucidate the possible association of high P2 flow with recurrent stroke risk in steno-occlusive disease of the anterior circulation.

### 4.4. Neurological Outcome

We show that poor intracranial flow status is associated with worse neurological status after recanalization, with lower M1 VFR ratios one week after stroke being associated with higher NIHSS scores at discharge. This was also true for the decrease in both M1 and total hemispheric VFR ratios between the early and late measurements. This finding might be linked to reperfusion failure, as persistent distal hypoperfusion following recanalization has been linked to long-term adverse outcomes and infarct growth [21,35,36,37,38]. These studies, however, performed imaging in an acute (<24 h) period after stroke, while in our study, early (<3 days) flow metrics were not significantly correlated with neurological outcome. This discrepancy might be partly explained by the use of perfusion imaging in the abovementioned studies, which may be more sensitive than arterial flow rates to early tissue hypoperfusion secondary to pathological microcirculation.

The relationship between early perfusion imaging after recanalization and the change in arterial flow rates over time remains unknown. It is possible that both parameters are highly correlated, with arterial flow decreasing in a subacute manner as a direct consequence of early distal reperfusion failure from microvasculature collapse. However, we cannot exclude other factors such as progressive oedematous swelling in larger infarcts, later microvascular dysfunction or haemorrhagic transformation, which can conceivably impact both arterial flow rates and neurological outcome, though we did not find significant associations between VFR changes over time and infarct size or haemorrhagic transformation.

It is important to note that the correlation between lower affected arterial flow and neurological outcome at 90 days no longer reached statistical significance (*p* = 0.079). This might be in part explained by the relatively small sample size of our study and the presence of further confounding variables (rehabilitation, social and financial status, comorbidities) which likely impact long-term neurological outcome. It is nonetheless worth noting that a recent study found a significant association between low post-treatment perfusion in the infarct core and penumbra, measured using intra-voxel incoherent motion imaging, and 90-day clinical outcome [38]. Further work will shed light on the relationship between the change in macrovascular flow over time and early distal perfusion status after recanalization.

It is perhaps likely that the relationship between M1 VFR ratio and neurological status is non-linear, i.e., that the benefit associated with a post-stroke flow ratio of the ischaemic vs. non-ischaemic hemisphere above 1 is less than the adverse effect of a flow ratio under 1. Indeed, in our study, all patients with NIHSS at discharge ≥ 5 showed an M1 VFR < 1. Further studies with larger sample sizes should explore this further.

### 4.5. Limitations

The limitations of this study include the relatively small sample size and single-centre patient cohort, limiting the generalizability of our results. Our statistical analysis was exploratory and hypothesis-generating, requiring confirmation in prospectively designed studies. Furthermore, we did not statistically adjust for systemic factors potentially affecting cerebral blood flow (such as extra- or intracranial atherosclerosis, haemorrhagic transformation, seizures, systemic infections, or the administration of cardiovascular drugs). Direct comparison with perfusion imaging was not performed, which did not allow the assessment of microcirculatory status. Finally, while the aim of this study was not to directly compare recanalized and non-recanalized patients, these presented with distinct stroke pathophysiology (recanalized MCA/ICA-MCA tandem occlusion vs. ICA occlusion likely secondary to chronic high-grade ICA stenosis).

## 5. Conclusions

Non-invasive qMRA may help to understand intracranial flow status and collateral pathway activation after LVO stroke. In this exploratory study, qMRA accurately reflected non-reperfusion through persistently low flow to the affected region. At week one, high P2 segment flow ratios of the ischaemic vs. non-ischaemic hemisphere were significantly correlated with low M1 segment flow ratios, possibly suggesting activation of secondary leptomeningeal collateral pathways. Persistent low arterial flow to the affected region past the acute phase was associated with poorer neurological status, including in patients successfully recanalized. Global intracranial arterial flow rates significantly decreased by up to 9% within the first week after LVO stroke, in both ischaemic and non-ischaemic hemispheres.

## Figures and Tables

**Table 1:** Patient inclusion and study flowchart.

	Recanalization		
Variable	Yes (*n* = 23)	No (*n* = 8)	*p*-Value
Age	67.5 (39–88)	60.1 (47–79)	0.20
Sex (male)	10	1	0.12
DWI infarct volume (mL)	48.7 (0.1–147.1)	14.0 (0.1–39.8)	0.001
Occluded vessel:			
ICA	7	8	<0.001
M1/M2	23	1	<0.001
Both	7	1	0.32
TOAST			
1	6	6	
2	11	0	
5	6	2	
ASITN/ISR			
0	2		
1	5		
2	8		
3	3		
4	2		
Comorbidities:			
Atrial fibrillation	7	0	0.08
Diabetes	2	2	0.24
Smoking	7	4	0.32
Hypertension	14	3	0.25
Dyslipidaemia	9	4	0.59
Obesity	3	0	0.28
Clinical status (median):			
NIHSS at admission	14 (1–20)	3 (0–11)	<0.001
mRS at admission	4 (1–5)	2 (0–4)	<0.001

## Data Availability

The raw data supporting the conclusions of this article will be made available by the authors on request.

## Supplementary Materials

The following supporting information can be downloaded at: https://www.mdpi.com/article/10.3390/brainsci16020171/s1. Table S1: linear model coefficients modelling NIHSS at discharge in recanalized patients.

## Abbreviations

The following abbreviations are used in this manuscript:

ACA	Anterior Carotid Artery
AComm	Anterior Communicating Artery
DSA	Digital Subtraction Angiography
DWI	Diffusion-Weighted Imaging
EVT	Endovascular Thrombectomy
ICA	Internal Carotid Artery
LVO	Large-Vessel Occlusion
MCA	Middle Carotid Artery
mRS	Modified Rankin Scale
NIHSS	National Institutes of Health Stroke Scale
NOVA	Non-Invasive Optimal Vessel Analysis
PCA	Posterior Carotid Artery
qMRA	Quantitative Magnetic Resonance Angiography
TOF	Time-of-Flight Angiography
VFR	Volumetric Flow Rate
