# Supplementary material for: Serial Intracranial Flow Rate Measurements Using Quantitative Magnetic Resonance Angiography Following Large-Vessel Occlusion Stroke
Source: Brain Sci. 2026 Jan 31;16(2):171. doi: 10.3390/brainsci16020171 (PMC12938040; doi:10.3390/brainsci16020171)
Supplement: Supplementary file 1 [file brainsci-16-00171-s001.zip › brainsci-4093188-supplementary.pdf]

# Supplementary Materials

| Predictor | Estimate (95% CI) | SE | p |
|---|---|---|---|
| Intercept | 8.81 (-1.33 to 18.95) | 4.61 | 0.082 |
| M1 VFR ratio at 7±3 days | -5.64 (-10.23 to -1.06) | 2.08 | 0.020 |
| NIHSS on admission | 0.30 (0.02 to 0.57) | 0.13 | 0.037 |
| DWI volume | 0.04 (-0.01 to 0.08) | 0.02 | 0.116 |
| Age | -0.05 (-0.14 to 0.03) | 0.04 | 0.206 |

***Supplementary Table S1:*** *linear model coefficients modelling NIHSS at discharge in recanalized patients. DWI, diffusion-weighted imaging; NIHSS, National Institutes of Health Stroke Scale.* $R^2$ *of model: 0.709*

Linear mixed models estimating the effects of time from stroke, recanalization status and hemisphere on absolute volumetric flow rates in each arterial segment took the form of:

Flow ~ 1 + NOVA_time + Treatment_group + Hemisphere + NOVA_time:Treatment_group + NOVA_time:Hemisphere + Treatment_group:Hemisphere + NOVA_time:Treatment_group:Hemisphere + ( 1 + Hemisphere | Patients )

Therefore, with patients as clustering variable, random intercepts were estimated for each patient, as well as a random effect of each patient on the difference in hemispheric volumetric flow.

Linear mixed models estimating the effects of scanning time and treatment group on volumetric flow rate ratios (ischemic/non-ischemic hemispheres) took the form of:

Ratios ~ 1 + NOVA_time + Treatment_group + NOVA_time:Treatment_group + ( 1 | Patients )

The VFR ratios were thus modelled as a random intercept-only model with patients as clustering variable.

## M1 VFR – LMM fixed effects estimates

| Names | Effect | Estimate | SE | 95% CI Lower | 95% CI Upper | df | t | p |
|---|---|---|---|---|---|---|---|---|
| (Intercept) | (Intercept) | 171.57 | 8.96 | 154.02 | 189.14 | 21.9 | 19.149 | < .001 |
| NOVA_time1 | NOVA_2 (4-10d) - NOVA_1 (<72h) | -15.39 | 8.58 | -32.21 | 1.41 | 40.7 | -1.795 | 0.080 |
| Treatment_group1 | Recanalized - Non-recanalized | 38.93 | 17.92 | 3.81 | 74.06 | 21.9 | 2.172 | 0.041 |
| Hemisphere1 | Non-affected - Affected | 29.96 | 11.16 | 8.09 | 51.83 | 21.5 | 2.684 | 0.014 |
| NOVA_time1 ✻ Treatment_group1 | NOVA_2 (4-10d) - NOVA_1 (<72h) ✻ Recanalized - Non-recanalized | -5.01 | 17.16 | -38.65 | 28.61 | 40.7 | -0.292 | 0.771 |
| NOVA_time1 ✻ Hemisphere1 | NOVA_2 (4-10d) - NOVA_1 (<72h) ✻ Non-affected - Affected | 0.06 | 16.92 | -33.10 | 33.24 | 41.8 | 0.004 | 0.997 |
| Treatment_group1 ✻ Hemisphere1 | Recanalized - Non-recanalized ✻ Non-affected - Affected | -50.40 | 22.32 | -94.15 | -6.66 | 21.5 | -2.258 | 0.034 |
| NOVA_time1 ✻ Treatment_group1 ✻ Hemisphere1 | NOVA_2 (4-10d) - NOVA_1 (<72h) ✻ Recanalized - Non-recanalized ✻ Non-affected - Affected | 28.78 | 33.85 | -37.55 | 95.12 | 41.8 | 0.850 | 0.400 |

## Random Components

| Groups | Name | SD | Variance | ICC |
|---|---|---|---|---|
| Patients | (Intercept) | 33.1 | 1098 | 0.488 |
| | Hemisphere1 | 30.6 | 939 | |
| Residual | | 33.9 | 1150 | |

Note. Number of Obs: 88, groups: Patients 23

## Random Components

| Groups | Name | SD | Variance | ICC |
|---|---|---|---|---|

## M1 VFR ratios – LMM fixed effects estimates

| | | | | 95% CI | | | | |
|---|---|---|---|---|---|---|---|---|
| **Names** | **Effect** | **Estimate** | **SE** | **Lower** | **Upper** | **df** | **t** | **p** |
| (Intercept) | (Intercept) | 0.8793 | 0.0595 | 0.7627 | 0.996 | 21.6 | 14.7700 | < .001 |
| NOVA_time1 | NOVA_2 (4-10d) - NOVA_1 (<72h) | -0.0401 | 0.0756 | 0.1883 | 0.108 | 20.5 | -0.5306 | 0.601 |
| Treatment_group1 | Recanalized - Non-recanalized | 0.2225 | 0.1191 | 0.0109 | 0.456 | 21.6 | 1.8686 | 0.075 |
| NOVA_time1 ✻ Treatment_group1 | NOVA_2 (4-10d) - NOVA_1 (<72h) ✻ Recanalized - Non-recanalized | 0.0126 | 0.1512 | 0.2837 | 0.309 | 20.5 | 0.0832 | 0.935 |

## Random Components

| Groups | Name | SD | Variance | ICC |
|---|---|---|---|---|
| Patients | (Intercept) | 0.194 | 0.0375 | 0.453 |
| Residual | | 0.213 | 0.0453 | |

Note. Number of Obs: 44, groups: Patients 23

## P2 VFRs – LMM fixed effects estimates

| Names | Effect | Estimate | SE | 95% CI | | df | t | p |
|---|---|---|---|---|---|---|---|---|
| | | | | Lower | Upper | | | |
| (Intercept) | (Intercept) | 84.996 | 5.05 | 75.09 | 94.90 | 21.0 | 16.8210 | < .001 |
| NOVA_time1 | NOVA_2 (4-10d) - NOVA_1 (<72h) | -10.591 | 3.34 | 17.13 | -4.05 | 42.0 | -3.1742 | 0.003 |
| Treatment_group1 | Recanalized - Non-recanalized | 1.743 | 10.11 | 18.06 | 21.55 | 21.0 | 0.1724 | 0.865 |
| Hemisphere1 | Non-affected - Affected | -1.125 | 4.60 | 10.15 | 7.90 | 21.0 | -0.2444 | 0.809 |
| NOVA_time1 ✻ Treatment_group1 | NOVA_2 (4-10d) - NOVA_1 (<72h) ✻ Recanalized - Non-recanalized | 0.652 | 6.67 | 12.43 | 13.73 | 42.0 | 0.0977 | 0.923 |
| NOVA_time1 ✻ Hemisphere1 | NOVA_2 (4-10d) - NOVA_1 (<72h) ✻ Non-affected - Affected | -7.123 | 6.67 | 20.20 | 5.96 | 42.0 | -1.0674 | 0.292 |
| Treatment_group1 ✻ Hemisphere1 | Recanalized - Non-recanalized ✻ Non-affected - Affected | 11.250 | 9.21 | -6.80 | 29.30 | 21.0 | 1.2219 | 0.235 |
| NOVA_time1 ✻ Treatment_group1 ✻ Hemisphere1 | NOVA_2 (4-10d) - NOVA_1 (<72h) ✻ Recanalized - Non-recanalized ✻ Non-affected - Affected | -2.578 | 13.35 | 28.74 | 23.58 | 42.0 | -0.1932 | 0.848 |

## Random components

| Groups | Name | SD | Variance | ICC |
|---|---|---|---|---|
| Patients | (Intercept) | 20.1 | 404 | 0.671 |
| | Hemisphere1 | 13.4 | 178 | |
| Residual | | 14.1 | 197 | |

Note. Number of Obs: 92, groups: Patients 23

## P2 VFR ratios – LMM fixed effects estimates

| | | | | 95% CI | | | | |
|---|---|---|---|---|---|---|---|---|
| **Names** | **Effect** | **Estimate** | **SE** | **Lower** | **Upper** | **df** | **t** | **p** |
| (Intercept) | (Intercept) | 1.0622 | 0.0518 | 0.9607 | 1.1637 | 21.0 | 20.510 | < .001 |
| NOVA_time1 | NOVA_2 (4-10d) - NOVA_1 (<72h) | 0.0971 | 0.0953 | -0.0897 | 0.2839 | 21.0 | 1.019 | 0.320 |
| Treatment_group1 | Recanalized - Non-recanalized | -0.1759 | 0.1036 | -0.3789 | 0.0271 | 21.0 | -1.698 | 0.104 |
| NOVA_time1 ✻ Treatment_group1 | NOVA_2 (4-10d) - NOVA_1 (<72h) ✻ Recanalized - Non-recanalized | -0.0457 | 0.1906 | -0.4193 | 0.3279 | 21.0 | -0.240 | 0.813 |

## Random Components

| Groups | Name | SD | Variance | ICC |
|---|---|---|---|---|
| Patients | (Intercept) | 0.0854 | 0.00730 | 0.0831 |
| Residual | | 0.2838 | 0.08055 | |

Note. Number of Obs: 46, groups: Patients 23

## A2 VFRs – LMM fixed effects estimates

| Names | Effect | Estimate | SE | 95% CI Lower | 95% CI Upper | df | t | p |
|---|---|---|---|---|---|---|---|---|
| (Intercept) | (Intercept) | 104.85 | 5.17 | 94.7 | 114.99 | 21.0 | 20.262 | < .001 |
| NOVA_time1 | NOVA_2 (4-10d) - NOVA_1 (<72h) | -7.66 | 4.70 | -16.9 | 1.55 | 42.0 | -1.630 | 0.111 |
| Treatment_group1 | Recanalized - Non-recanalized | 9.36 | 10.35 | -10.9 | 29.65 | 21.0 | 0.905 | 0.376 |
| Hemisphere1 | Non-affected - Affected | 2.64 | 11.51 | -19.9 | 25.19 | 21.0 | 0.229 | 0.821 |
| NOVA_time1 ✻ Treatment_group1 | NOVA_2 (4-10d) - NOVA_1 (<72h) ✻ Recanalized - Non-recanalized | 5.68 | 9.40 | -12.7 | 24.10 | 42.0 | 0.604 | 0.549 |
| NOVA_time1 ✻ Hemisphere1 | NOVA_2 (4-10d) - NOVA_1 (<72h) ✻ Non-affected - Affected | -8.54 | 9.40 | -27.0 | 9.89 | 42.0 | -0.908 | 0.369 |
| Treatment_group1 ✻ Hemisphere1 | Recanalized - Non-recanalized ✻ Non-affected - Affected | 4.61 | 23.01 | -40.5 | 49.71 | 21.0 | 0.200 | 0.843 |
| NOVA_time1 ✻ Treatment_group1 ✻ Hemisphere1 | NOVA_2 (4-10d) - NOVA_1 (<72h) ✻ Recanalized - Non-recanalized ✻ Non-affected - Affected | -17.75 | 18.80 | -54.6 | 19.11 | 42.0 | -0.944 | 0.351 |

## Random Components

| Groups | Name | SD | Variance | ICC |
|---|---|---|---|---|
| Patients | (Intercept) | 19.4 | 377 | 0.490 |
| | Hemisphere1 | 44.2 | 1957 | |
| Residual | | 19.8 | 392 | |

Note. Number of Obs: 92, groups: Patients 23

## A2 VFR ratios – LMM fixed effects estimates

| | | | | 95% CI | | | | |
|---|---|---|---|---|---|---|---|---|
| **Names** | **Effect** | **Estimate** | **SE** | **Lower** | **Upper** | **df** | **t** | **p** |
| (Intercept) | (Intercept) | 1.0144 | 0.1088 | 0.8012 | 1.228 | 21.0 | 9.323 | < .001 |
| NOVA_time1 | NOVA_2 (4-10d) - NOVA_1 (<72h) | 0.1033 | 0.0717 | -0.0372 | 0.244 | 21.0 | 1.441 | 0.164 |
| Treatment_group1 | Recanalized - Non-recanalized | 0.0458 | 0.2176 | -0.3807 | 0.472 | 21.0 | 0.211 | 0.835 |
| NOVA_time1 ✻ Treatment_group1 | NOVA_2 (4-10d) - NOVA_1 (<72h) ✻ Recanalized - Non-recanalized | 0.1819 | 0.1434 | -0.0991 | 0.463 | 21.0 | 1.269 | 0.218 |

## Random Components

| Groups | Name | SD | Variance | ICC |
|---|---|---|---|---|
| Patients | (Intercept) | 0.433 | 0.1872 | 0.804 |
| Residual | | 0.213 | 0.0456 | |

Note. Number of Obs: 46, groups: Patients 23

## Hemispheric VFR – LMM fixed effects estimates

| | | | | 95% CI | | | | |
|---|---|---|---|---|---|---|---|---|
| **Names** | **Effect** | **Estimate** | **SE** | **Lower** | **Upper** | **df** | **t** | **p** |
| (Intercept) | (Intercept) | 362.81 | 16.9 | 329.67 | 396.0 | 21.5 | 21.453 | < .001 |
| NOVA_time1 | NOVA_2 (4-10d) - NOVA_1 (<72h) | -33.44 | 11.3 | -55.50 | -11.4 | 39.3 | -2.971 | 0.005 |
| Treatment_group1 | Recanalized - Non-recanalized | 50.25 | 33.8 | -16.04 | 116.5 | 21.5 | 1.486 | 0.152 |
| Hemisphere1 | Non-affected - Affected | 33.19 | 16.1 | 1.69 | 64.7 | 21.6 | 2.065 | 0.051 |
| NOVA_time1 ✻ Treatment_group1 | NOVA_2 (4-10d) - NOVA_1 (<72h) ✻ Recanalized - Non-recanalized | 6.87 | 22.5 | -37.24 | 51.0 | 39.3 | 0.305 | 0.762 |
| NOVA_time1 ✻ Hemisphere1 | NOVA_2 (4-10d) - NOVA_1 (<72h) ✻ Non-affected - Affected | -17.26 | 22.1 | -60.65 | 26.1 | 41.0 | -0.779 | 0.440 |
| Treatment_group1 ✻ Hemisphere1 | Recanalized - Non-recanalized ✻ Non-affected - Affected | -36.21 | 32.1 | -99.21 | 26.8 | 21.6 | -1.126 | 0.272 |
| NOVA_time1 ✻ Treatment_group1 ✻ Hemisphere1 | NOVA_2 (4-10d) - NOVA_1 (<72h) ✻ Recanalized - Non-recanalized ✻ Non-affected - Affected | 15.33 | 44.3 | -71.46 | 102.1 | 41.0 | 0.346 | 0.731 |

## Random components

| Groups | Name | SD | Variance | ICC |
|---|---|---|---|---|
| Patients | (Intercept) | 67.2 | 4512 | 0.698 |
| | Hemisphere1 | 49.1 | 2408 | |
| Residual | | 44.2 | 1955 | |

Note. Number of Obs: 88, groups: Patients 23

## Hemispheric VFR ratios – LMM fixed effects estimates

| | | | | 95% CI | | | | |
|---|---|---|---|---|---|---|---|---|
| **Names** | **Effect** | **Estimate** | **SE** | **Lower** | **Upper** | **df** | **t** | **p** |
| (Intercept) | (Intercept) | 0.9251 | 0.0409 | 0.8449 | 1.005 | 21.5 | 22.611 | < .001 |
| NOVA_time1 | NOVA_2 (4-10d) - NOVA_1 (<72h) | 0.0289 | 0.0514 | -0.0718 | 0.130 | 20.5 | 0.562 | 0.580 |
| Treatment_group1 | Recanalized - Non-recanalized | 0.0906 | 0.0818 | -0.0697 | 0.251 | 21.5 | 1.108 | 0.280 |
| NOVA_time1 ✻ Treatment_group1 | NOVA_2 (4-10d) - NOVA_1 (<72h) ✻ Recanalized - Non-recanalized | -0.0132 | 0.1028 | -0.2146 | 0.188 | 20.5 | -0.129 | 0.899 |

## Random Components

| Groups | Name | SD | Variance | ICC |
|---|---|---|---|---|
| Patients | (Intercept) | 0.134 | 0.0180 | 0.462 |
| Residual | | 0.145 | 0.0209 | |

Note. Number of Obs: 44, groups: Patients 23
